# Dietary

**Published:** 1891-01

**Authors:** 


### DIETARY.

Physicians always order beef for invalids that is cooked very little, in order that none of the nourishment in the meat may be dried away. Lean beef ground in a machine, salted to taste, made into cakes, and broiled just enough to heat, is excellent for invalids to whom the doctor has forbidden vegetables. A person in health may suit his taste.

Eat all cold food slowly. Digestion will not begin till the temperature of the food has been raised by the heat of the stomach to ninety-eight degrees. Hence the more heat that can be imparted to it by slow mastication the better. The precipitation of a large quantity of cold in the stomach by fast eating, may, and often does, cause discomfort and indigestion, and every occasion of this kind results in a measurable injury to the digestive functions. Ice water drunk with cold food of course increases the mischief. Hot drinks—hot water, weak tea, coffee, chocolate, etc.—will, on the contrary, help to prevent it. But eat slowly, any way.

A famous doctor says : " Eat a good bowl of mush and milk for your breakfast, and you will not need any medicine. Indian corn contains a large amount of nitrogen, has qualities anti-constipating, and is easily assimilated. It is cheap and has great nutritive properties. A course of Indian meal in the shape of Johnny-cake, hoe-cake, corn or pone bread and mush, relieved by copious draughts of pure cow's milk, to which, if inclined to dyspepsia, a little lime water may be added, will make a life now a burden well worth the living, and you need no other treatment to correct your nervousness, brighten your vision, and give you sweet and peaceful sleep."

Rev. Mark Trafton says : " I am to-day as straight in my spinal column as a pine of my native State. At the age of 20 I was in the itinerant ministry of the Methodist Episcopal Church, and when I had been preaching two years a physician said to me : 'You must stop preaching or you will not live five years.' He has been in his grave 40 years ; after this busy and exciting life of 60 years, I am here writing a word to my coevals, and 'my eye is not dim, nor my natural force (much) abated.' Why? Because, with the blessing of God, I have watched the operation of nature's teaching and obeyed the teacher, and taken care of myself. For eight or nine years past I have eaten no flesh of dead animals. For many years I have eaten whole wheat or Graham bread. My breakfast is the principal meal for the day—two soft boiled eggs, a saucer of oatmeal, mush, bread, and one cup of coffee. My dinner is bread, a slice or two, a cup of weak tea; at night, a half a pint of milk and a slice of bread. I hardly know, from any sensation, whether I have eaten or not. I have gained in weight, and suppose, unless some accident befall me, or I slip into some indiscretion, I shall be at last a centenarian."

